# Impact of heat stress, water stress, and their combined effects on the metabolism and transcriptome of grape berries

**DOI:** 10.1038/s41598-023-36160-x

**Published:** 2023-06-19

**Authors:** Seanna Hewitt, Esther Hernández-Montes, Amit Dhingra, Markus Keller

**Affiliations:** 1 Department of Horticulture, Washington State University, Pullman, WA USA; 2 Department of Viticulture and Enology, Irrigated Agriculture Research and Extension Center, Washington State University, Prosser, WA USA; 3 Department of Agricultural Production, CEIGRAM, Universidad Politécnica de Madrid, Madrid, Spain; 4 Department of Horticultural Sciences, Texas A&M University, College Station, TX USA

**Keywords:** Plant physiology, Plant stress responses, Plant molecular biology

## Abstract

Recurring heat and drought episodes present challenges to the sustainability of grape production worldwide. We investigated the impacts of heat and drought stress on transcriptomic and metabolic responses of berries from two wine grape varieties. Cabernet Sauvignon and Riesling grapevines were subjected to one of four treatments during early fruit ripening: (1) drought stress only, (2) heat stress only, (3) simultaneous drought and heat stress, (4) no drought or heat stress (control). Berry metabolites, especially organic acids, were analyzed, and time-course transcriptome analysis was performed on samples before, during, and after the stress episode. Both alone and in conjunction with water stress, heat stress had a much more significant impact on berry organic acid content, pH, and titratable acidity than water stress. This observation contrasts with previous reports for leaves, which responded more strongly to water stress, indicating that grape berries display a distinct, organ-specific response to environmental stresses. Consistent with the metabolic changes, the global transcriptomic analysis revealed that heat stress had a more significant impact on gene expression in grape berries than water stress in both varieties. The differentially expressed genes were those associated with the tricarboxylic acid cycle and glyoxylate cycle, mitochondrial electron transport and alternative respiration, glycolysis and gluconeogenesis, carbohydrate allocation, ascorbate metabolism, and abiotic stress signaling pathways. Knowledge regarding how environmental stresses, alone and in combination, impact the berry metabolism of different grape varieties will form the basis for developing recommendations for climate change mitigation strategies and genetic improvement.

## Introduction

Grapevine (*Vitis* spp.) is a valuable and versatile fruit crop worldwide, primarily grown in temperate regions characterized by warm and dry summers. Grape berries are used in producing juice, wine, distilled liquor, table grapes, and raisins^1^. In addition to its commercial diversity and high economic value, the grapevine has emerged as a model perennial fruit crop for the study of drought and heat tolerance, with many of a diverse array of cultivated varieties adapted to grow in temperate regions with Mediterranean climates^2,3^.

The rising temperature and declining water availability associated with global climate change pose challenges for high-quality wine grape production, especially in already warm and dry regions. Known effects of high temperature on grape quality include undesirable decreases in key chemical constituents like organic acids and anthocyanins and increases in sugar, leading to higher alcohol contents in wines^4,5^. Organic acids affect the organoleptic properties in wines, such as the perception of acidity and freshness, while also contributing to microbial stability during wine storage. Higher temperatures cause a general reduction of malic acid in grapes at maturity, with negative consequences for wine quality. However, the accumulated malic acid in grapes is consumed by respiration and gluconeogenesis processes throughout fruit maturation, and these processes are temperature-sensitive^6^. By comparison, temperature effects on the other major organic acid in grapes, tartaric acid, are largely unknown. This example underlies the importance of intensifying the study of temperature-dependent pathways involved in berry primary metabolism and the genes associated with these pathways during berry ripening.

Hand-in-hand with increasing temperature is an increased prevalence of drought conditions. Moderate water deficit can benefit grape production, promoting the accumulation of desirable metabolites such as anthocyanins and other phenolics^7–10^. Although the use of irrigation is becoming more common, a majority of grape-growing regions remain unirrigated. In those regions, the plants and berries may be exposed to periods of drought as part of their regular growing regime^2^. Severe and prolonged heat and drought episodes can be detrimental to wine grape production, hindering growth, negatively impacting yield and fruit metabolism, and on a global scale, impacting the distribution of winegrowing areas^11,12^. Even irrigated grape production regions are experiencing the impacts of climate change, with mountain snowpacks becoming less reliable as temperatures continue to rise^13^.

Adapting wine grape production to changing climatic conditions will require varietal diversification and integration of traits that improve resilience to heat and drought stress^3,5^. Further, identifying physiological, metabolic, and genetic factors contributing to abiotic stress tolerance and grape berry composition is urgently needed. While the effects of water stress and heat stress on grapevine leaf physiology have been well characterized^14–21^, there is still much to be learned about the impacts of these stress factors—particularly in conjunction with one another—on the physiology, metabolism, and underlying gene expression in grape berries^22^. Several diverse hypotheses have been advanced to explain the roles of abiotic stresses on grape development. One such hypothesis is that water stress directly alters fruit metabolism via the influence of root-derived or locally produced abscisic acid (ABA) or via auxin signaling ^23,24^. Another hypothesis states that variety-specific differences in hydraulic behavior translate into differences in the metabolic response of grape berries to water stress^25^. Alternatively, water stress may indirectly affect grape composition by reducing canopy size and density, which increases sun exposure of the fruit clusters and hence berry temperature^16^. A hybrid model of both the direct and indirect effects of water stress on berry metabolism and associated gene expression has also been proposed^26,27^. Thus, it remains unclear whether the presumed water stress effect on berry composition is direct or whether it is mediated by berry temperature (and light). The interaction between water deficit and temperature is expected to impact biological pathways associated with the production of key metabolites for wine production, such as sugars, organic acids, anthocyanins, and other phenolics, as well as diverse aroma volatiles that contribute to the flavor and quality of the wine^2,22^.

By testing the effects of drought and heat stress alone or in combination, this study sought to fill the knowledge gap in understanding the individual and interactive effects of water deficit and temperature on grape berry metabolism and underlying gene expression. We used pot-grown plants in climate-controlled growth chambers to avoid the effect of solar heating of grape berries under water stress that is inevitable under field conditions. We assayed key metabolites and conducted a global gene expression analysis in ripening berries of two contrasting wine grape varieties, Cabernet Sauvignon and Riesling. Overall, this study revealed genes and associated pathways involved in grape berry abiotic stress response and how these pathways vary under heat and drought stress by variety. Knowledge regarding how grapevines respond to the interaction between heat and drought stress and how grape ripening is impacted will form the basis for developing enhanced recommendations for irrigation management and grapevine genetic improvement in the context of climate change.

## Materials and methods

### Plant material and experimental conditions

The experiment was carried out under controlled environmental conditions at the Irrigated Agriculture Research and Extension Center in Prosser, Washington, USA, in 2018 using two-year-old, own-rooted Riesling and Cabernet Sauvignon grapevines (*Vitis vinifera* L.). The plants were sourced from Clean Plant Center Northwest, Washington State University, Prosser, WA. Plants were grown in 20-L pots filled with a mix of 50% (v/v) sandy loam soil, 25% peat moss, and 25% pumice; in addition, 3 g L^−1^ of dolomite was added to the mix. A complete slow-release fertilizer was applied at the six-leaf stage, at anthesis (beginning of bloom), and after fruit set. Vines were pruned to four shoots and thinned to one fruit cluster per shoot. Plants were grown outdoors until the first berries started to soften (9 August), then they were moved to four 11.3-m^2^ growth chambers (TPRB-111, BioChambers, Winnipeg, MB, Canada) for acclimatization. During the 6-day acclimatization period, vines were irrigated daily to 100% of field capacity. Each growth chamber was equipped with temperature and light control. Chamber lighting consisted of 50% metal-halide lamps (186–204, Venture, Solon, OH, USA) and 50% high-pressure sodium-vapor lamps (HPS ET18, Sylvania, Danvers, MA, USA), providing a maximum photosynthetic photon flux of 1100 µmol m^−2^ s^−1^ at canopy height.

After the acclimatization period, four treatments were applied for 7 days: heat stress (HS), water stress (WS), heat and water stress (HWS), and no stress or control (C). Cabernet Sauvignon and Riesling vines (6 single-vine replicates per treatment) from the C and WS treatments were randomly assigned to two growth chambers. Vines from the HS and HWS treatments were randomly assigned to the other two chambers to account for possible chamber effects. Light conditions and temperature were programmed to change hourly to simulate the photoperiod and average hourly temperature registered in the field for the veraison (beginning of ripening) period of the two varieties from 2006 to 2016. The temperatures in the control treatment group changed diurnally from 15 °C (3:00–5:00) to 29 °C (13:00–16:00), and the relative humidity changed from 75 to 40%. In the heat stress chambers, hourly temperatures were increased by 10 °C relative to the control. Relative humidity in the HS chambers ranged from 50 to 25%. Irrigation treatments were established by measuring soil water content using a 20-cm TDR probe (HydroSense II, Campbell Scientific, Logan, UT, USA) and weighing the pots daily. Well-watered vines were irrigated daily to field capacity, and water-stressed vines were irrigated to approximately 50% of field capacity. After the stress period, a 7-day recovery period followed, using the control temperature schedule and well-watered conditions.

All methods were performed on grape plants that were cultivated for the purposes of the experiments, including the collection of plant material for all analysis, and all relevant institutional, national, and international guidelines and legislation were complied with.

### Berry sample collection

Independent berry samples were collected for metabolite and transcriptomic analysis. Ten berries per plant were randomly collected for chemical analysis on day 7 of the stress period. Additionally, five berries per plant were randomly collected for transcriptomic analysis before the stress period started (day 0, ‘Before Stress’), on day 7 of the stress period (‘During Stress’), and on day 7 of the recovery period (‘After Stress’). All samples were collected into zip-lock plastic bags, flash-frozen in liquid nitrogen, and stored at − 80 °C.

### Metabolite analysis

Berries were thawed at room temperature and weighed using a precision balance (AX205 DR, Mettler-Toledo, Greifensee, Switzerland), and juice was extracted manually by pressing the berries inside the plastic bags. The juice total soluble solids (TSS) concentration was analyzed using a benchtop refractometer (MT RE40D, Mettler-Toledo). The juice was stored in 15-mL centrifuge tubes at − 20 °C for organic acid analysis. Grape juice was thawed at room temperature, heated to 71 °C for 20 min^28^, and cooled to room temperature. Titratable acidity (TA) was analyzed to an end-point of pH 8.1 using an auto-titrator (Titrino plus 848, Metrohm, Herisau, Switzerland) connected to a compact sample changer (869 CSC, Metrohm). The pH was measured using an MP225 Quattro pH meter (Mettler-Toledo). The juice was centrifuged at 13,250 *g* for 15 min and filtered through a 0.45-µm membrane (Nanosep® Centrifugal Filters). The juice was diluted to analyze organic acids using an Agilent 7100 capillary electrophoresis system (Agilent, Santa Clara, CA, USA) following the protocol of the Agilent Organic Acids Solution kit (PN 5063–6510). Berry metabolite data were analyzed by three-way analysis of variance (ANOVA) using SAS University Edition (SAS Institute, Cary, NC, USA) to test for effects of temperature, water, variety, and their interactions.

### RNA extraction and sequencing

Grape berries (seeds removed) were pulverized under liquid nitrogen using a mortar and pestle. Total RNA was extracted from three biological replicates, each replicate comprised of homogenate of 6 berries each, using a modified DEPC-CTAB protocol^29^, with three technical replicates performed for each biological replicate at the ‘Before Stress’, ‘During Stress’, and ‘After Stress’ time points. RNA was quality-checked on an agarose gel and was quantified using a Nanodrop 2000 spectrophotometer (Thermo Fisher Scientific, Waltham, MA, USA). Following quality validation and quantification using a Qubit Fluorometer (Thermo Fisher Scientific) and Agilent Bioanalyzer (Santa Clara, CA, USA), cDNA libraries were prepared from the RNA and sequenced by BGI Genomics (Hong Kong, China) on an Illumina HiSeq 4000 platform as 2 × 150 paired-end reads.

### Transcriptome assembly and treatment read mapping

The 2 × 150 paired-end fastq files generated using Illumina HiSeq 4000 were input into the CLC Genomics Workbench (ver. 8.5.1) for pre-processing and assembly according to published methods^30,31^. Briefly, the CLC Create Sequencing QC report tool was used to assess the quality and determine the amount of sequence to trim. The CLC Trim Sequence process was then used to trim quality scores with a limit of 0.001, corresponding to a Phred value of 30. The 13, 5′ terminal nucleotides were removed, as were any ambiguous nucleotides. Reads below length 51 were discarded. Overlapping pairs were merged using the ‘Merge Overlapping Pairs’ tool, following which a de novo assembly was performed with all trimmed, merged datasets. The de novo assembly resulted in the production of 195,894 contiguous sequences (contigs). Contigs with less than 2 × coverage and those less than 200 bp in length were eliminated. For each dataset (treatment/replicate), the original, non-trimmed reads were mapped back to the master assembly contig subset. Mapping resulted in the generation of individual treatment sample reads per contig. The master transcriptome was exported as a fasta file for functional annotation, and the read counts for each dataset were exported for normalization with the Reads Per Kilobase per Million reads (RPKM) method^32^. Contigs with RPKM values of less than 0.5 for all treatments and time points were filtered out. The final working dataset consisted of 87,867 contigs.

### Differential expression analysis

Differentially expressed genes for each treatment, in comparison with the control, were identified using the time course, multi-series differential expression feature in the OmicsBox suite, which employs the maSigPro R package. The FDR-corrected cutoff was *p* ≤ 0.05. The statistical analysis ensured that genes that did not meet the assumption of equal variances were eliminated from the analysis. The differentially expressed genes (DEGs) and expression values were matched with their corresponding functional annotations (Supplementary File 1).

### Functional annotation

The master transcriptome fasta file produced from the Illumina assembly was imported into OmicsBox 1.4.11 (BioBam Bioinformatics S.L., Valencia, Spain) for functional annotation of expressed contigs. Contig sequences were identified by a blastx alignment against the NCBI ‘Viridiplantae’ database with an e-value specification of 10.0E-3f. GO annotation was assigned using the ‘Mapping’ and ‘Annotation’ features using default parameters to generate a functionally annotated master assembly^33^.

### Gene ontology enrichment analysis

GO enrichment analysis using Fisher’s exact test was conducted in OmicsBox to identify the cellular components, molecular functions, and biological processes that were over- or under-represented in the various stress treatments in comparison with the control berries for both grape varieties (FDR-corrected *p*-value < 0.001). Lists of the differentially expressed, functionally annotated genes were generated for the Cabernet Sauvignon and Riesling during HS, WS, and HWS. These lists served as the treatment datasets for enrichment analyses, and the master annotated transcriptome was used as the reference dataset. Prior to conducting enrichment analysis, the Go-Slim feature was used to reduce the number of GO terms present in the annotated reference transcriptome to overarching functions and processes displaying the greatest enrichment.

## Results and discussion

### Leaf physiology

In conjunction with this study, we recently reported the impact of drought stress and heat stress in the same experiment on shoot growth, leaf physiological traits, abscisic acid (ABA) and proline levels, and expression of key genes involved in ABA and proline biosynthesis in leaves^21^. Water availability dominated the growth and leaf physiological responses, while heat stress only played a minor role, even with daily maximum leaf temperatures close to 40 °C. Compared with the control, WS significantly reduced shoot elongation, leaf water potential (Ψ_l_), stomatal conductance (g_s_), photosynthesis, and transpiration, while strongly increasing leaf ABA. By contrast, with the exception of Ψ_l,_ heat stress rarely altered any of these physiological traits and did not impact shoot growth, though it tended to exacerbate the effect of water stress on leaf physiology. Riesling leaves seemed to be somewhat more sensitive to heat stress, especially in combination with water stress than were Cabernet Sauvignon leaves. For example, based on the decrease in g_s_, Riesling vines experienced severe water stress (g_s_ < 0.05 mol H_2_O m^−2^ s^−1^) under both WS and HWS, but Cabernet Sauvignon only reached extreme stress under HWS^21^. In both varieties, however, midday Ψ_l_ decreased from an average of − 0.8 MPa in the control to − 1.2 MPa under HS to -1.3 MPa under both WS and HWS. While sufficiently low to induce stomatal closure, these values are still high enough for grapevines to avoid xylem cavitation and leaf wilting^2^. All vines were exposed to the stress treatments for 7 days; thus, the mature leaves on which our measurements were conducted had developed under non-stress conditions. These results confirm recent data obtained using Malbec grapevines and show that leaves are relatively resilient with respect to heat stress, at least in the temperature range (≤ 40 °C) tested here^17^. Earlier work had found the optimum temperature for photosynthesis of grape leaves to be in the range of 25‒30 °C^34^.

### Berry metabolites

**Figure 1:**
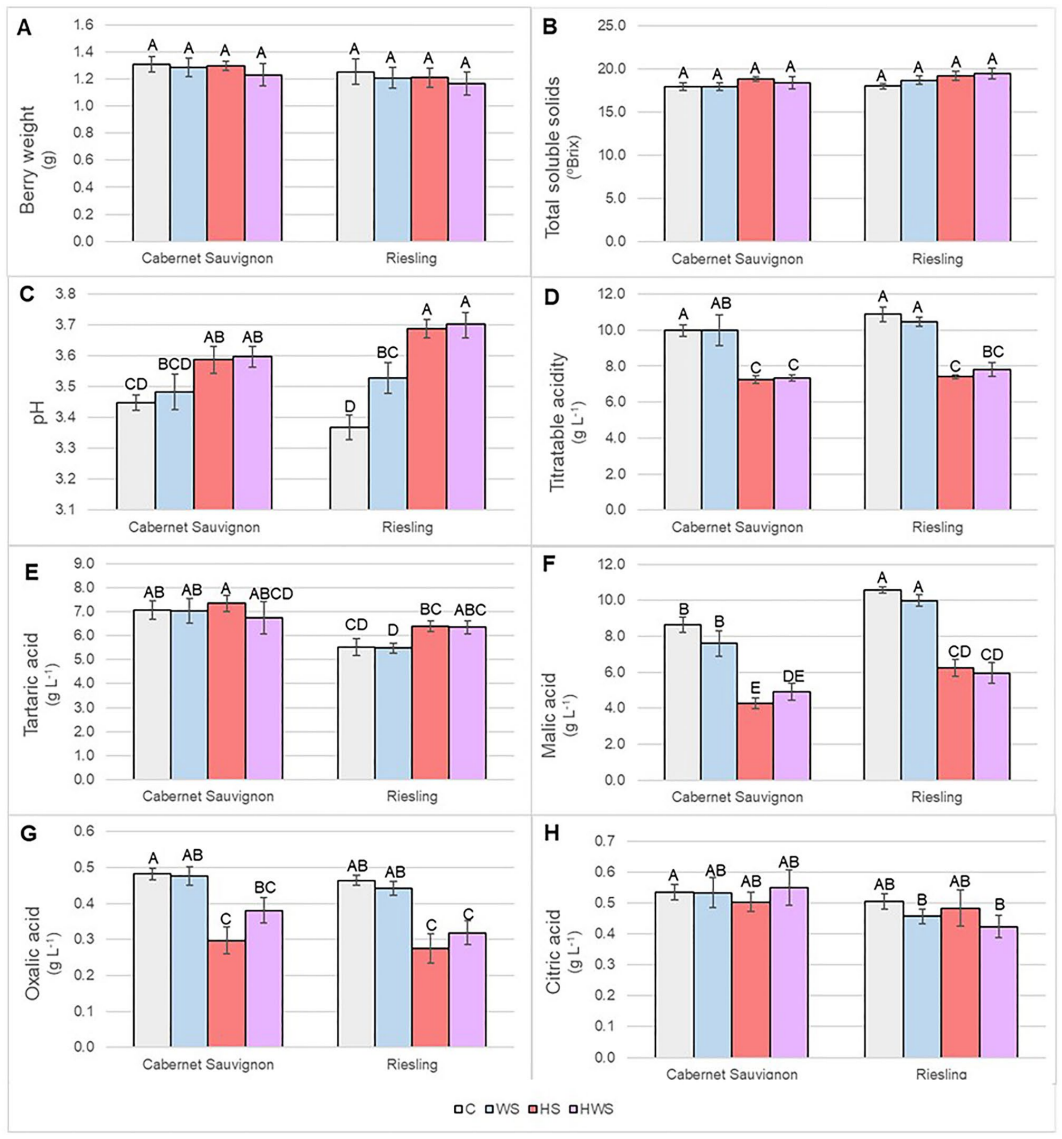
Effects of heat stress (HS), water stress (WS), and heat + water stress (HWS) at the beginning of fruit ripening on Cabernet Sauvignon and Riesling grape berry composition. Control (C) values are also shown for reference. Parameters measured included (**A**) berry weight, (**B**) total soluble solids, (**C**) pH, (**D**) titratable acidity, (**E**) tartaric acid, (**F**) malic acid, (**G**) oxalic acid, and (**H**) citric acid. Different letters indicate significant differences (*p* < 0.05). Error bars represent standard error (n = 6).

In sharp contrast to most leaf physiological traits and shoot growth^21^, the variation in berry metabolite composition was dominated by the effect of temperature, while water availability played, at most, a minor role. The different measures of fruit composition in the berries collected after 7 days of stress (Fig. 1) indicated that these berries were in the middle of the ripening period, during which sugars accumulate rapidly and malic acid is degraded^35^. Despite the up to 70% reduction in leaf photosynthesis under WS^21^, neither berry weight nor TSS (which is a robust proxy for sugar concentration in ripening grapes) were significantly impacted by any of the stress treatments (Fig. 1A and B). Similar results were found in a heat-stress experiment with Cabernet Sauvignon grapes^36^ and Muscat Hamburg grapes^37^ and in a water-stress experiment with different grape varieties^38^. In another study, however, in which Sémillon grapes were exposed to 40/25 °C day/night temperatures for 4 days at the beginning of ripening, heat stress inhibited both berry growth and sugar accumulation^39^. Therefore, the effect of heat stress might depend on the variety or the way treatments are applied. For example, while our study applied realistic, diurnal irradiance and temperature profiles, many other growth chamber studies used static day/night conditions. In both of our varieties, heat stress increased the berry pH (Fig. 1C). In Riesling, the WS berries had an intermediate pH (Fig. 1C). Heat stress (HS and HWS) decreased TA by 26–32%, malic acid (MalA) by 40–52%, and oxalic acid (OxA) by 20–42% in both varieties (Fig. 1D), while water stress by itself had no effect (Fig. 1E). Among organic acids, MalA is metabolized during grape ripening, while OxA continues to accumulate^40^. Two other organic acids, tartaric acid (TartA) and citric acid (CitA) displayed variety-specific responses with regard to the impact of stress on their accumulation. TartA, like OxA a derivative of ascorbic acid (vitamin C) metabolism^22^, was not significantly impacted in Cabernet Sauvignon under any stress condition but was elevated in Riesling under heat stress (Fig. 1G). CitA was not significantly impacted by any stress condition in either variety (Fig. 1H).

In practice, the impact of elevated temperature during the growing season often requires acid addition to adjust wine acidity and reduce the pH to appropriate levels, an endeavor that can incur significant expenses. Unlike MalA, which is easily metabolized under heat stress^41,42^, TartA is much more stable at high temperatures^6,35^. In our study, heat stress during the early ripening phase slightly increased TartA in Riesling but not Cabernet Sauvignon berries. In contrast, Lecourieux et al.^36,43^ reported an increase in Cabernet Sauvignon berries exposed to elevated temperature (~ 34 °C vs. 26 °C at veraison). It is conceivable that there may be varietal differences in the activation of antioxidative processes in an effort to maintain homeostasis under heat stress. Despite the limited response of TartA to temperature, the lower acidity of grape berries that accompanied exposure of grapevines to HS and HWS, but not WS, indicates that temperature has a particularly pronounced effect on fruit acidity (especially MalA and OxA), which is not brought about by water deficit alone. This is consistent with the hypothesis that the effects of water stress on grape composition, or at least on organic acid metabolism, may be indirect and mediated directly by temperature (and light), arising from a decrease in shoot growth under water stress and the associated increase in fruit exposure to sunlight, causing solar heating of the berries^16^. Though water stress decreased shoot growth in our study as well^21^, we excluded solar heating as a complicating variable by applying temperature treatments inside environmentally-controlled growth chambers. Unlike in many field experiments, moreover, leaf senescence (yellowing and abscission) was not observed with the treatment structure used in our study.

In summary, whereas water stress had a direct effect on leaf physiological processes (which may be mediated by hydraulic properties as well as hormones such as ABA), it did not affect berry organic acid metabolism in a similar way. The latter, instead, was much more strongly impacted by temperature. It is likely that the pronounced effect of temperature in grape berries compared with leaves is a consequence of the low transpiration rate and, thus, the poor evaporative cooling capacity of grape berries^44^. During ripening, moreover, water is supplied to the berries via the phloem rather than the xylem, so the berries are relatively well buffered against soil water deficit^38,45^.

### Transcriptome assembly and annotation

Functional annotation of the 87,867 contigs from the final grape assembly dataset resulted in the assignation of blast results to 63,032 (71.73%) of contigs and functional annotation to a total of 55,182 contigs (62.80%) (Supplementary File 1).

### Differentially expressed genes and enriched gene ontologies

**Figure 2:**
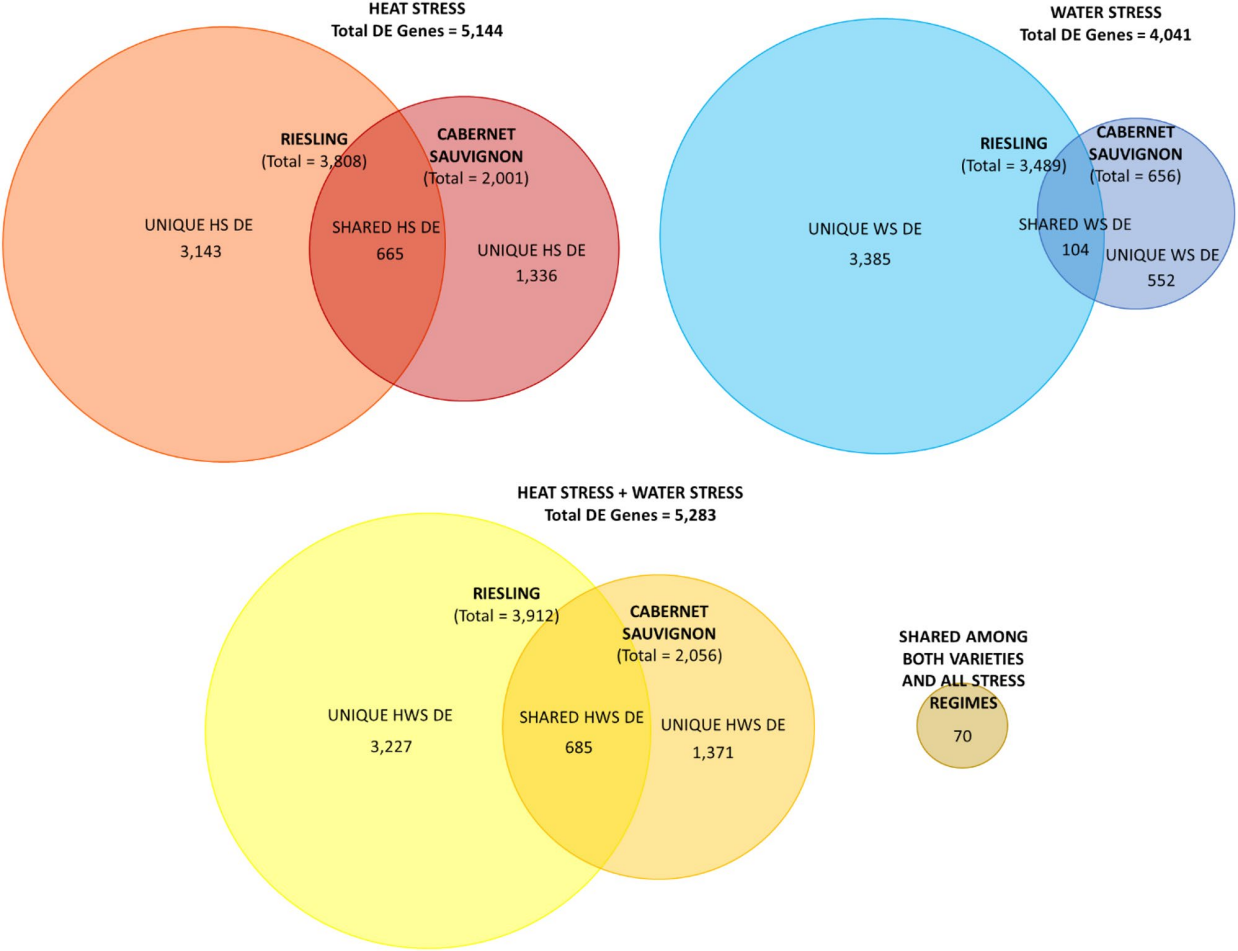
Numbers of genes that were significantly differentially expressed over time (*p* < 0.05) compared to the control in berries of Cabernet Sauvignon and Riesling grapevines exposed to heat stress (HS), water stress (WS), or combined heat and water stress (HWS) at the beginning of fruit ripening. A further subset of 70 genes was identified among the shared HS, WS, and HWS DE genes—these were differentially expressed in all stress regimes in both grape varieties.

A total of 5144; 4041; and 5283 annotated genes were significantly differentially expressed over time in HS, WS, and HWS treatments, respectively. Of these, 2001 HS, 656 WS, and 2056 HWS genes were differentially expressed in Cabernet Sauvignon, and 3,808 HS, 3498 WS, and 3912 HWS genes were differentially expressed in Riesling (Fig. 2). In addition, a set of 70 genes that displayed shared differential expression across all treatments and in both grape varieties was identified—these were classified as “core stress genes” (Fig. 2, Supplementary File 2).

In Cabernet Sauvignon, gene expression response was consistent with the berry metabolite results, with the highest number of DEGs identified in the HWS and HS treatments, while WS elicited fewer expression changes in comparison with HS and HWS. Riesling displayed more DE genes in all treatments compared to Cabernet Sauvignon; this was particularly notable in the berries exposed to the WS treatment. Cabernet Sauvignon displayed fewer DEGs than Riesling, and this was particularly notable in the berries exposed to the WS treatment. The greater number of DEGs identified in Riesling (Fig. 2), and the elevated number of genes impacted by WS in this variety, suggests a heightened sensitivity of Riesling berries to both temperature and water stress compared to Cabernet Sauvignon, consistent with the physiological observations in leaves^21^.

Gene ontology enrichment analysis was conducted using lists of DE genes identified in HS, WS, and HWS treatments for each variety (6 lists total). Core stress ontologies enriched for both varieties in all stress treatments include GOs corresponding to transcription factor activity and gene expression regulation (GO:0003700, GO:0140110, GO:0003723, GO:0008135, GO:0090079); primary cellular metabolic processes, including the metabolism of carbohydrates, lipids, and organic substances (GO:0044238, GO:0005975, GO:0006629, GO:0006807, GO:0071704, GO:0006091); response to stresses and stimuli (GO:0006950, GO:0009607, GO:0042221); signal transduction (GO:0007165, GO:0023052, GO:0038023, GO:0060089, GO:0016301); enzymatic regulation of biological processes (GO:0065007, GO:0030234, GO:0003824, GO:0065009), transporter activity (GO:0006810, GO:0005215); and maintenance of cellular homeostasis (GO:0019725). Several shared ontologies enriched in both genotypes during the HS and HWS treatments, but not WS, included a response to abiotic stimulus (GO:0009628) and cell growth/differentiation (GO:0030154, GO:0040007). Additionally, several ontologies associated with the development of shoot and reproductive structures (GO:0048367, GO:0048608, GO:0061458) were enriched in both varieties during WS; however, during HS and HWS, these were only enriched in Riesling. Similarly, secondary metabolic processes (GO:0019748) were enriched for both varieties in the HWS treatment but only for Riesling in the HS and WS treatments. Compared to variety and stress-specific ontologies, the large number of shared enriched ontologies suggests that a common group of pathways is activated in response to heat and water stress in Cabernet Sauvignon and Riesling grape berries.

While the majority of the enriched ontologies identified were variety and stress agnostic, there were a few GOs identified that were enriched in a variety-specific manner. In Cabernet Sauvignon, chromatin binding (GO:0003682) and protein-containing complex binding (GO:004487) were enriched in the HS and HWS treatments only. In Riesling, unique GOs enriched in all stress treatments included circadian rhythm (GO:0007623) and response to radiation (GO:0009314); unique GOs enriched in HS and WS (but not HWS) included terms associated with epigenetic regulation of gene expression (GO:0006338, GO:0010468, GO:0040029); and unique GOs enriched in WS and HWS (but not HS) included carbohydrate binding (GO:0030246) (Table 1).

**Table 1:**
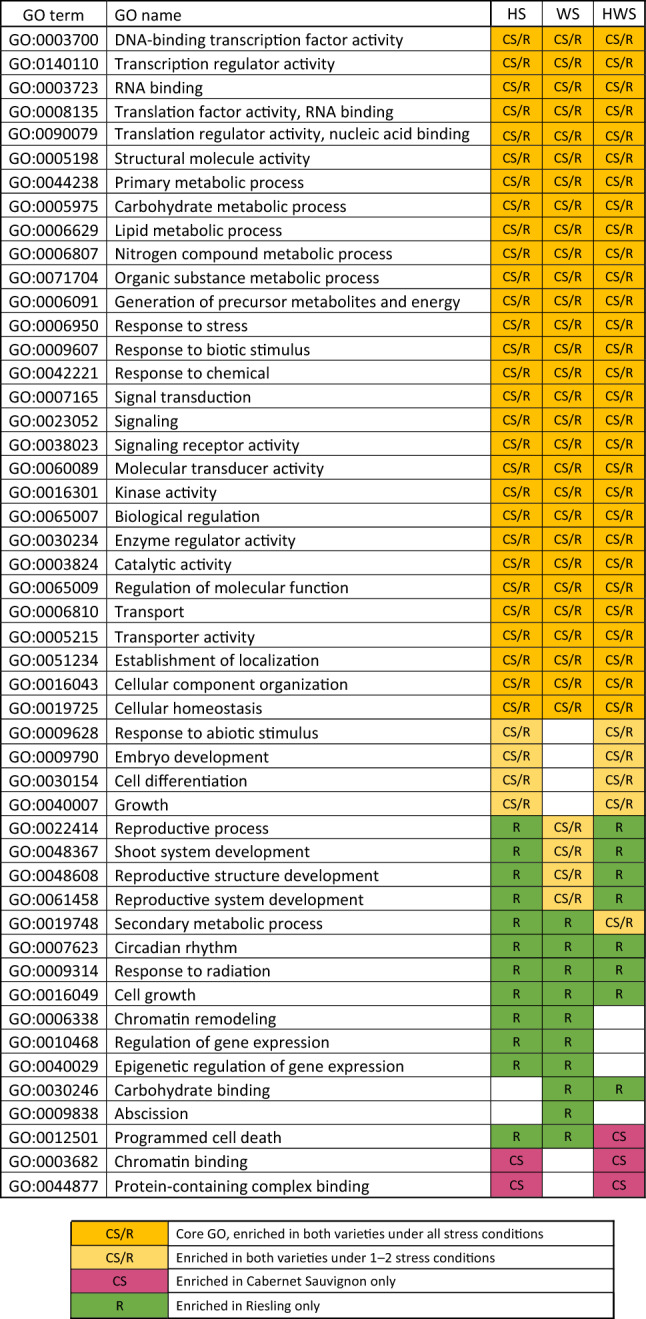
Enriched gene ontology (GO) terms in Cabernet Sauvignon (CS) and Riesling (R) grape berries exposed to heat stress (HS), water stress (WS), or heat + water stress (HWS).

While the number and nature of the differentially expressed genes varied by treatment and stress type—with more variety- and stress-specific genes identified than shared DE genes (Fig. 2)—interestingly, these diverse gene sets corresponded to highly similar enrichment results. This finding suggests that the berries of the two varieties seem to adopt different strategies at the gene expression level when exposed to individual or concomitant stress to activate different aspects of stress-responsive pathways and ultimately achieve similar biological outcomes. Overall, the enriched ontologies indicate a global stress response involving enhanced gene expression regulation, stress signal transduction, energy production, and primary metabolic activities.

Due to the correspondence with the TSS and organic acid assays performed, the pathways corresponding to gene ontologies ‘organic substance metabolic process’, ‘carbohydrate metabolic process,’ ‘primary metabolic process,’ ‘generation of precursor metabolites and energy’ were investigated in greater detail at the level of individual genes involved in the corresponding pathways. Additionally, to better understand the observation that variety and stress-specific gene expression patterns underly similar enriched gene ontologies, the pathways corresponding to transcription factor activity and stress signal transduction are also explored further, as the signaling-associated genes are expected to lend insight into variety and stress regime-specific initial response to heat and drought.

### Organic acid metabolism

Organic acid content is a critical component of fruit and wine organoleptic quality. Most acids accumulate until grape berries undergo a metabolic shift at the onset of ripening. However, acid content, and therefore TA and pH, are highly influenced by genotype and environmental conditions^46^. High-temperature-driven reduction of organic acids, particularly malate, has been studied primarily in red grapevine varieties, including Shiraz^46^, Cabernet Sauvignon^43,47^, Muscat Hamburg^37^, and DCRF mutants (microvine)^42^. Recently, the effects of combined temperature and drought stress on two white varieties, Chardonnay and Xynisteri, were also studied^48^. Results of these studies and the present work suggest that WS and HS may impact grape berries of different varieties in different ways, with the metabolic and physiological responses much more pronounced under heat stress.

**Figure 3:**
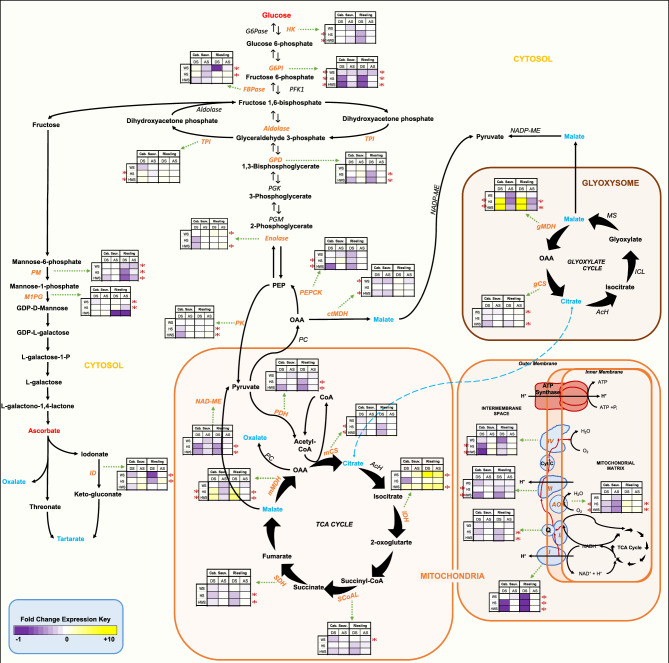
Organic acid metabolism (TCA cycle and glyoxylate cycle), mitochondrial electron transport, glycolysis/gluconeogenesis, and ascorbate metabolic pathways in berries of Cabernet Sauvignon and Riesling grapevines subjected to water stress (WS), heat stress (HS), or heat + water stress (HWS) at the beginning of fruit ripening. Bold blue text indicates organic acids assayed in this study: malic acid (MalA)/malate, citric acid (CitA)/citrate, oxalic acid (OxA)/oxalate, tartaric acid (TartA)/tartrate. Bold/italicized orange text indicates enzymes corresponding to significantly differentially expressed genes: organic acid metabolism/TCA cycle—pyruvate dehydrogenase (PDH), NAD malic enzyme (NAD-ME), mitochondrial malate dehydrogenase (mMDH), cytosolic malate dehydrogenase (ctMDH), mitochondrial citrate synthase (mCS), mitochondrial isocitrate dehydrogenase (mIDH), succinyl-CoA ligase (SCoAL), succinate dehydrogenase (SDH); mitochondrial electron transport—Complex I (NADH-uibquinone oxidoreductase), Complex II (succinate dehydrogenase), Complex III (Ubiquinol-cytochrome-c reductase), Complex IV (Cytochrome c oxidase), and alternative oxidase (AOX); glyoxylate cycle—glyoxysomal malate dehydrogenase (gMDH), glyoxysomal citrate synthase (gCS); glycolysis/gluconeogenesis—hexokinase (HK), glucose-6-phosphate isomerase (G6PI), fructose-1,6-bisphosphatase (FBPase), aldolase, triosephosphate isomerase (TPI), glyceraldehyde-3-phosphate dehydrogenase (GPD), enolase, pyruvate kinase (PK), phosphoenolpyruvate carboxykinase (PEPCK); ascorbate metabolism—phosphomannomutase (PM), mannose-1-phosphate guanylyltransferase (M1PG), and iodonate dehydrogenase (ID). Red arrows in mETC indicate the direction of electron flow. Dashed green arrows link DEGs to their corresponding expression heatmaps. Heatmap boxes show the expression of significant DEGs as fold change of each treatment during and after stress (DS and AS, respectively) normalized to the before stress control condition. Red asterisks indicate significant (*p* < 0.05) differential expression over time (as assessed via MaSigPro) for Cabernet Sauvignon (left side of heatmaps) and Riesling (right side of heatmaps).

Several of the assayed organic acids are directly (MalA, CitA) or indirectly (OxA) associated with the TCA cycle and the glyoxylate cycle. The former pathway is responsible for the breakdown of pyruvate produced during glycolysis, the generation of donor molecules for mitochondrial electron transport and substrates for other metabolites, and the production of CO_2_, while the glyoxylate cycle is involved in the breakdown of fatty acids to produce substrates for gluconeogenesis. Significantly elevated expression of a DEG encoding the mitochondrial isoform of malate dehydrogenase (mMDH), which catalyzes the conversion of MalA to oxaloacetic acid (OAA), was observed in HS and HWS grapes, corresponding to reduced levels of MalA in the berries (Fig. 3). Glyoxysomal malate dehydrogenase (gMDH) also displayed high transcript abundance during stress. In contrast to MDH isoform expression, most other TCA and glyoxylate cycle enzyme-encoding genes displayed reduced expression during both HS and WS and especially under HWS (Fig. 3). The enzyme in the TCA cycle downstream of mMDH, citrate synthase (mCS), displayed reduced transcript abundance during WS and HWS in Cabernet Sauvignon, and during HS and HWS in Riesling, with significant changes in mCS expression only observed in Riesling. In the glyoxysome, expression of gCS displayed a similar pattern in Cabernet Sauvignon and Riesling during HWS; after stress, however, expression increased significantly in Riesling WS and HWS (Fig. 3). The expression changes in CS isoforms during stress corresponded to reduced CitA levels in Riesling, while no significant change in CitA accumulation was observed in Cabernet Sauvignon.

The third metabolite assayed, OxA, can be produced via a number of pathways, including decarboxylation of OAA and metabolism of ascorbate and/or glyoxylate (Fig. 3)^49^. Assuming a hypothetical, unidirectional production of TCA and glyoxylate cycle metabolites, the increased MDH and decreased CS transcript abundance would be expected to indicate a greater flux of OAA into the production of OxA. However, this is not what we observed in this study, as OxA levels decreased significantly in both Cabernet Sauvignon and Riesling under heat stress (Fig. 3), suggesting an alternative fate of OAA, such as a gluconeogenic precursor or biomineralization to calcium-oxalate^50,51^.

Venios et al.^20^ described a comprehensive physiological response of grape berries to heat stress, characterized by reduced TA, reduced MalA, increased sugar-to-acid ratio, reduced flavanol, and anthocyanin production, and increased sugar content. The described compositional changes are consistent with those measured in the present study, particularly with regard to TA and MalA content. However, while previous studies have sought to elucidate the effects of heat on different stages of berry development and in different temperature regimes^46,52,53^, the present study explored the different effects that the interaction of heat and water stress have in both red and white grape varieties during the early ripening phase.

### Carbohydrate metabolic processes—glycolysis and gluconeogenesis

It has been suggested that transcriptome remodeling in response to high temperature disrupts the synchrony of sugar and organic acid metabolism during grape berry development^42^. Consistent with this idea, our study found heat stress effects on organic acids but not sugar in ripening grape berries (Fig. 1). Thus, in conjunction with the assessment of organic acid metabolism at the transcriptome level, it was also of interest to observe expression patterns of genes involved in gluconeogenesis as well as glycolysis. Gluconeogenesis has a temperature optimum near 20 °C, decreasing to half the maximum rate at 30 °C^54^. Thus, the effects of elevated temperature are expected to elicit a particularly notable effect on gluconeogenic processes.

Hexokinase, the first committed enzyme in glycolysis, displayed a significant reduction in transcript abundance in Riesling during HS and HWS in comparison with the control; however, in Cabernet Sauvignon, no significant change in expression of this gene was observed under any of the stress conditions (Fig. 3). A similar trend was observed for the second step in the glycolytic pathway, phosphohexose isomerase, where significant decreases in gene expression were measured during WS, HS, and HWS in berries of both grape varieties, particularly at the peak of stress treatment.

### Mitochondrial electron transport

In general, reduced expression of genes associated with mitochondrial ETC complexes I, II, III, and IV was observed during stress in both varieties. In most cases, HS and HWS had the greatest impact on the reduction of transcriptional activity. Transcription associated with alternative respiration (AOX homolog, ubiquinol oxidase 2) was elevated in Riesling but repressed in Cabernet Sauvignon—the AOX gene is activated in response to stress and plays a role in the reduction of ROS^55^. The heightened expression in Riesling may be indicative of an elevated ROS scavenging response in this variety. As Riesling berries, unlike those of Cabernet Sauvignon, do not accumulate anthocyanins, they might utilize an alternative strategy by which to mitigate oxidative stress, although both varieties displayed elevated expression of ROS scavenging genes in response to HS and HWS (Fig. 3).

### Heat and water stress signal transduction pathways

In the general model for heat stress response in grape berries^20^, stress sensor proteins respond to elevated temperatures and transmit heat stress signals via ROS and secondary messengers that activate signal transducers, such as mitogen-activated protein kinases (MAPKs), that further relay the stress signal. This activates a transcriptional network comprised of stress-related proteins and chaperones (e.g., heat shock proteins [HSPs], heat stress transcription factors [HSFs]; ascorbate peroxidase [APX], and dehydroascorbate reductase [DHAR]) that ultimately confers tolerance to heat stress.

**Figure 4:**
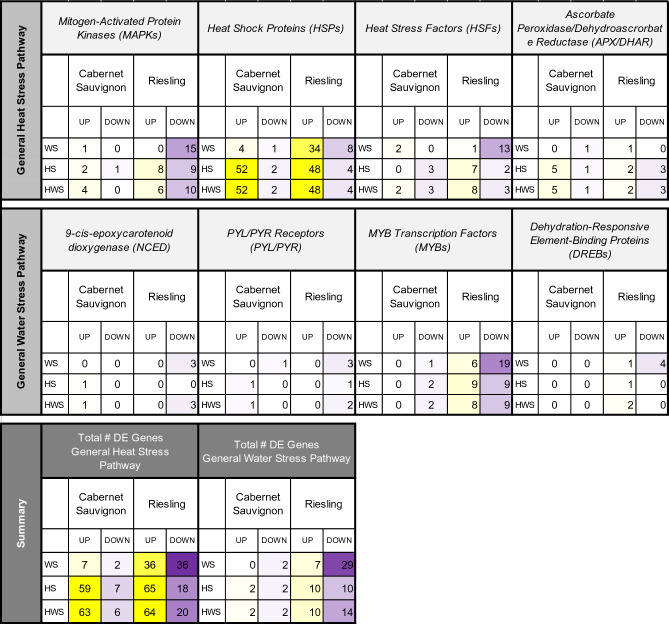
Number of general heat stress pathway and general water stress pathway genes that displayed a significant expression trend (as determined by MaSigPro) over time in comparison with the control in Cabernet Sauvignon and Riesling grape berries exposed to water stress (WS), heat stress (HS), and/or heat + water stress (HWS) treatments. Yellow indicates elevated expression, and purple indicates decreased expression, with the brightness of the color corresponding to the number of DE genes (darker = more DE genes). The total number of DE genes by treatment and grape variety is summarized at the bottom.

In both Cabernet Sauvignon and Riesling, several *MAPK* genes, numerous *HSP* genes, and several *HSF*, *APX*, and *DHAR* genes displayed significant responses to HS and HWS treatments (Fig. 4, top). Unique to Riesling, many *HSP*s were significantly upregulated under WS as well, indicating these transcription factors may also play a role in the WS response, in addition to the HS response, of this variety. Several significant *MAPK*s were identified in both varieties, with more significant DEGs observed in Riesling, as well as a greater balance of upregulated to downregulated genes. Though Cabernet Sauvignon displayed fewer significant *MAPK* genes, nearly all were upregulated. *HSP*s, not surprisingly, represented a substantial portion of significant DEGs pertaining to the general heat stress pathway (Fig. 4, top). Both Cabernet Sauvignon and Riesling had high numbers (i.e., 52 and 48) of upregulated DEGs in the HS and HWS treatment. As with *MAPKs,* Riesling displayed a high number of upregulated *HSP*s under WS, suggesting that HSPs play a role in the WS response in this variety (Fig. 4, top). In addition to the *MAPK* and *HSP* primary signal transducers, differentially expressed *HSF*s were identified in both varieties, with stronger representation in Riesling. Multiple differentially expressed *APX* and *DHAR* genes were identified in both varieties, with a higher representation of upregulated DEGs in Cabernet Sauvignon HS and HWS treatments and a greater balance of upregulated and downregulated DEGs observed in Riesling. The greater balance of upregulated to downregulated genes in Riesling is suggestive of a more fine-tuned mechanism of stress response regulation, which may be necessary in the absence of pigmented antioxidants, like anthocyanins, that accumulate in red varieties. This is in contrast to Cabernet Sauvignon, which displayed fewer differentially expressed genes overall but for which a greater percentage of the DEGs were upregulated under HS and HWS treatments (Fig. 4, top).

In the general model for response to water deficit^56^, drought response may be activated in both ABA-dependent and independent manners. In the case of the former, water deficit triggers the accumulation of ABA and ABA receptor activity (e.g., increased expression of *NCED*, *PYR*, and *PYL*), which leads to activation of *MYB/MYC* transcription factors, which together coordinate the hormone-mediated drought response. In the case of the latter, drought conditions activate dehydration-responsive binding elements and cold-binding factors (*DREB*s and *CBF*s), which coordinate the water stress response by *MYC*, *MYB*, and other transcription factors in a stress hormone-independent manner.

This study revealed that Cabernet Sauvignon berries displayed significant temporal upregulation of ABA receptor-encoding *NCED* and *PYL/PYR* genes in HS and HWS but not WS (Fig. 4, middle), which contrasts with the response observed in leaves^21^. No significantly upregulated DEGs encoding ABA receptors were observed for Riesling, and conversely, all of the differentially expressed genes identified for this class were downregulated (primarily in the WS treatment). *MYB/MYC* genes were represented among the DEGs for both varieties; however, no significantly upregulated *MYB/MYC* DEGs were observed in Cabernet Sauvignon, and a larger number of these transcription factors, as well as a greater balance of upregulated and downregulated genes, was observed in Riesling (Fig. 4, middle). Significant *DREB* genes were not represented in Cabernet Sauvignon at all, while Riesling displayed differential expression of *DREB* genes for all stress treatments (Fig. 4, middle). Taken together, these findings suggest that, unlike their leaves, the berries of both grape varieties likely respond to water stress in an ABA-independent manner, although ABA-dependent mechanisms may be at play in the heat stress response of Cabernet Sauvignon. Moreover, the heightened number of significant DEGs for the WS treatment, as well as the greater balance of upregulated and downregulated genes, in Riesling indicates that the berries of this variety may be more sensitive to water stress than those of Cabernet Sauvignon, and therefore may require more fine-tuned regulation of its responses (Fig. 4, bottom).

## Conclusion

The results of this study highlight the effects of temperature and water availability and the combined effects of heat and water stress on grape berry metabolism and composition. Exposure to a 7-day episode of heat stress, and heat in combination with water stress, during early grape ripening, increased berry pH, decreased TA, and generally decreased organic acid content, but did not alter sugar content, in the berries of two distinct wine grape varieties. The finding that heat stress was more impactful than water stress at the metabolic level was paralleled at the transcriptome level in Cabernet Sauvignon but not Riesling. Moreover, we identified important differences in comparing the global and transcriptomic responses of Cabernet Sauvignon and Riesling berries to the different kinds of stresses, as well as the variation in responses of key metabolic and stress-responsive pathways including glycolysis and gluconeogenesis, the TCA and glyoxylate cycles, mETC and AOX, abscisic acid metabolism, and general heat and water stress response pathways. A more pronounced response to water stress was observed for Riesling berries in comparison with Cabernet Sauvignon berries at the transcriptome level, with a greater number of significantly differentially expressed genes and a greater balance of genes that were upregulated versus downregulated. Cabernet Sauvignon, on the other hand, displayed substantially fewer differentially expressed genes overall (~ 1/2 that of Riesling in HS, ~ 1/5 that of Riesling in WS, and ~ 1/2 that of Riesling in HWS [Fig. 2]), with a majority of the general heat and water stress pathway genes significantly upregulated in expression during stress. The similar berry metabolite-level responses of the two varieties, in conjunction with the notable differences in gene expression of key metabolic and stress-responsive pathways underlying similar enriched gene ontologies, suggests that these two varieties may have somewhat different genetic mechanisms for mitigating water stress and heat stress, alone or in combination, that ultimately result in a similar physiological outcome. The﻿ goal of this study was to investigate the short-term effects of heat stress, water stress, and the combined effects of both on berry metabolism and gene expression. Moving forward, the identification of physiological and genetic factors that contribute to both short and long-term abiotic stress tolerance of different grape varieties is of particular importance to devising viticultural and genetic strategies for the mitigation of abiotic stresses.

## Supplementary Information

Supplementary Information 1.

Supplementary Information 2.

## Data Availability

The RNAseq datasets generated during the current study are available in the Short Read Archive on the NCBI database. The accession number is PRJNA928668, can be accessed here: https://www.ncbi.nlm.nih.gov/bioproject/PRJNA928668.
